# Effectiveness and safety of Arecae Semen compounds for patients with depression: a systematic review and meta-analysis

**DOI:** 10.1186/s13643-024-02656-4

**Published:** 2024-09-19

**Authors:** Tong Lin, Xiaoyu Zang, Yi Chen, Linhua Zhao, Ying Zhang

**Affiliations:** 1 Institute of Integrated Traditional Chinese and Western Medicine, Beijing University of Chinese Medicine, Beijing, 100029 China; 2 Institute of Metabolic Diseases, Guang’anmen Hospital, China Academy of Chinese Medical Sciences, Beijing, 100053 China; 3 Graduate School of Changchun University of Chinese Medicine, Changchun, Jilin Province 130117 China; 4 The First School of Clinical Medical Sciences, Guangzhou University of Chinese, Guangzhou, 510405 China; 5 Center for Evidence-based Chinese Medicine, Beijing University of Chinese Medicine, Beijing, 100029 China

**Keywords:** Arecae Semen, Traditional Chinese medicine, Depression, Systematic review, Meta-analysis

## Abstract

**Background:**

Arecae Semen is a traditional herbal medicine widely used in the medical service and food industry, but in recent years, the carcinogenesis of edible Arecae Semen chewing has aroused comprehensive attention, therefore it is necessary to evaluate its medicinal properties. Increasing evidence has shown that Arecae Semen Compounds (ASC) possess antidepressant ability. This study aimed to evaluate the effectiveness and safety of ASC in the treatment of depression.

**Methods:**

We retrieved articles in eight databases from their inception to May 2024. Randomized controlled trials (RCTs) comparing the effects of ASC alone or combined with routine treatment in patients with depression were identified. The Cochrane risk of bias (ROB) tool (ROB 2) was used for assessing the ROB in the included trials. Grading of Recommendations Assessment, Development, and Evaluation (GRADE) was used to assess the certainty of the evidence for the review outcomes. The outcomes included Hamilton depression rating scale (HAMD) scores, depression-related symptoms, serum dopamine levels, and adverse events. Stata 14.0 was used for data analysis calculating standardized mean difference (SMD) for continuous outcomes and relative risk (RR) for binary outcomes, both with 95% confidence intervals (CI).

**Results:**

Nine RCTs involving 787 patients were included in this review. ASC lowered HAMD scores (SMD − 3.43, 95% CI − 5.24 to − 1.61; *I*^2^ = 95.2%, *P* < 0.001), alleviated depression-related symptoms, increased serum dopamine levels, and reduced the incidence of adverse events slightly (RR 0.18, 95% CI 0.04 to 0.77; *I*^2^ = 0, *P* = 0.775) compared with the control group. Publication bias might account for the asymmetrical presentation of funnel plots. Meta-regression analysis revealed that regarding HAMD scores, there was no significant relationship with duration, sample size, or treatment strategy. The evidence of the outcomes was of very low certainty.

**Conclusions:**

ASC may achieve better therapeutic effects, alleviate depression-related symptoms with a lower incidence of adverse events, and provide a potentially effective and safe complementary therapy for patients with depression. However, the evidence is very uncertain so further researches are required to validate our results and explore clinical implications of Arecae Semen in depth.

**Systematic review registration:**

PROSPERO CRD42022361150.

**Supplementary Information:**

The online version contains supplementary material available at 10.1186/s13643-024-02656-4.

## Introduction

Depression is a prevalent mental disorder characterized by depressed mood, discouragement, lack of interest, feelings of guilt, sleep disorders, and a series of somatic symptoms. According to reports released by the World Health Organization in 2021, approximately 5% of adults suffer from depression, which has become a global public health problem [1]. Depression is projected to be one of the most important causes of the burden of disease by 2030 and severe cases may pose a potential threat to life [2].

Currently, clinical practice follows the principle of individualized treatment, and modern medicine is mainly treated with antidepressant drugs, assisted by various treatment methods such as psychotherapy [3]. Current pharmacological agents for depression mainly include selective serotonin reuptake inhibitors (SSRIs), serotonin-norepinephrine reuptake inhibitors (SNRIs), tricyclic antidepressants, and so on, which are widely used to alleviate depressive symptoms [4]. The side effects of traditional antidepressants such as nausea, insomnia, dry mouth, and emotional numbing, often influence medication compliance, therefore patients are prone to discontinue the treatments [5]. These interventions with long-term use attain lower remission rates and lead to weight gain, metabolic dysfunction, and tardive dyskinesia with long-term use [6]. Traditional Chinese medicine (TCM) is also constantly exploring better clinical interventions for depression. At present, there are countless clinical studies on the treatment of depression with TCM therapy [7, 8]. Increasing researches have proved that TCM therapy can produce the pharmacological effects of regulating the hypothalamic–pituitary–adrenal axis, preventing dysfunctional hippocampal neurogenesis, and ameliorating depression-like behaviors [9–11]. Although the mechanism hasn’t been fully elucidated, the TCM treatment of depression shows certain advantages in effectiveness and safety [12, 13].

Arecae Semen a critical traditional herbal medicine grows in much of Asia, the tropical Pacific, and parts of East Africa. Edible Arecae Semen is processed into dried betel nut for chewing consumption and primarily sold to Pakistan, Indonesia, Hunan, and Hainan provinces in China. Arecae Semen has been widely used in clinical practice in the form of TCM compounds, including Yueju Baohe Pill, Binglang Shisanwei Pill, and Muxiang Shunqi Decoction, as examples, and has been proven to have antidepressant pharmacological active ingredients [14, 15]. Years of clinical experience have proved that Arecae Semen possesses antidepressant ability. Recently, the carcinogenesis of Arecae Semen has aroused comprehensive attention, its pharmacological effects require further exploration. From the perspective of TCM, Arecae Semen compounds (ASC) have the effects of dispersing stagnated liver qi for relieving qi stagnation, which is consistent with the pathogenesis of depression. A number of randomized controlled studies on the treatment of depression by ASC have been published, showing that TCM monotherapy or combined with antidepressant drugs has curative effects and fewer adverse reactions [16, 17]. Therefore, as informed by the TCM theory, ASC is a critical treatment for depression and the investigation of ASC may provide new value for depression treatment. To explore whether ASC alone or ASC combined with routine treatment alleviates depression, we reviewed randomized controlled trials (RCTs) that assessed the effectiveness and safety of these ASCs for antidepressant effects and adverse events compared with routine treatment, providing a complementary therapy for depressive patients.

## Methods

### Search strategy

We retrieved articles from the following databases: PubMed, Cochrane Library, Embase, Web of Science, Chinese National Knowledge Infrastructure, Wanfang Database, Chinese Biomedical Database (CBM), and China Science and Technique Journals Database (VIP) with the use of Medical Subject Headings (MeSH) terms from their inception to May 2024 without language restriction. The keyword terms “Arecae Semen”, “traditional Chinese medicine”, and “depression” were used in the initial search. Search strategies for English and Chinese databases are presented in Tables 1 and 2 correspondingly.

**Table 1:** Search strategies for English databases

Databases	Search strategy
PubMed	#1 ((Areca[Title/Abstract) OR (Areca[Mesh])) OR (Simo Decoction[Title/Abstract])) OR (Simo Decoction[Mesh]) #2 (depression[Title/Abstract]) OR (depression[Mesh]) #3 #1 AND #2
Cochrane Library	#1 (Areca) OR (Areca catechu) OR (Arecae Semen) OR (Betel) OR (Betel Nut) OR (Simo Decoction) #2 (depression):ti,ab,kw OR (depressive disorder):ti,ab,kw OR (depressive symptom):ti,ab,kw #3 #1 AND #2
Embase	#1 'areca' OR 'areca catechu' OR 'arecae semen' OR 'betel' OR 'betel nut' OR ‘Simo Decoction’ #2 'depression':ab,kw,ti OR 'depressive disorder':ab,kw,ti OR 'depressive symptom':ab,kw,ti #3 #1 AND #2
Web of Science	#1 ALL = (Areca OR Areca catechu OR Arecae Semen OR Betel OR Betel Nut OR Simo Decoction) #2 TS = (depression OR depressive disorder OR depressive symptom) #3 #1 AND #2

**Table 2:** Search strategies for Chinese databases

Databases	Search strategy
CNKI	(SU = '槟榔' OR TKA = '槟榔' OR TI = '槟榔' OR KY = '槟榔' OR AB = '槟榔' OR FT = '槟榔') AND SU = ('抑郁症' + '抑郁')
Wanfang Database	(主题:(槟榔) or 全部:(槟榔)) and (主题:(抑郁症) or 主题:(抑郁))
CBM	("槟榔"[标题] OR "槟榔"[摘要] OR "槟榔"[全部字段]) AND ("抑郁症"[标题] OR "抑郁症"[摘要] OR "抑郁"[标题] OR "抑郁"[摘要])
VIP	(M = 槟榔 OR R = 槟榔 OR U = 槟榔) AND (M = (抑郁症 OR 抑郁) OR R = (抑郁症 OR 抑郁))

*SU* subject, *TKA* title, keyword, abstract, *TI* title, *KY* keyword, *AB* abstract, *FT* full text

### Eligibility and exclusion criteria

Only RCTs for evaluating the effectiveness of ASC on depression were included in this review. Patients included in the present review met the following criteria: (1) diagnosed with depression according to the Chinese Classification and Diagnostic Criteria of Mental Disorders (Third Edition) (CCMD-3) [18] or the Diagnostic and Statistical Manual of Mental Disorders (Fifth Edition) (DSM-5) criteria [19]; (2) no restriction on gender, and with an adult age from 18 to 80; (3) trial participants gave written informed consent. The following studies were excluded: (i) patients suffered from cognitive impairment chronic or other major psychiatric disorders; (ii) none of the review’s pre-specified outcomes were reported; (iii) the studies were duplicate publications, expert experience, conference references, or non-RCTs. There was no restriction on sex or region. Patients in the control group received routine treatment, such as antidepressants and usual care. The intervention group was treated with ASC alone or combined with routine treatment regardless of the treatment duration. The primary outcomes were Hamilton depression scale (HAMD) scores, and the secondary outcomes were depression-related symptoms, serum dopamine levels, and adverse events (including headache, abdominal discomfort, and fatigue).

### Study selection and data extraction

Two reviewers screened the titles and abstracts independently based on the inclusion and exclusion criteria and then scanned full articles to identify eligible trials. Discrepancies were resolved through discussion with a third reviewer. The Preferred Reporting Items for Systematic Reviews and Meta-Analyses (PRISMA) flowchart was used to record the study selection process (Additional file 1).

The data were extracted using the self-designed form by two reviewers independently. The basic information included author, year, age, sample size, intervention, follow-up, outcome measures, and adverse events. Discrepancies were discussed through consultation with a third reviewer.

### Assessing the risk of bias

The risk of bias in included trials was assessed by two researchers independently using the Cochrane risk of bias (ROB) tool (ROB 2) [20], which contained the following aspects: allocation sequence generation, allocation concealment, blinding, missing outcome data, and selective outcome reporting. For each item, the response options were “definitely yes”, “probably yes”, “probably no”, and “definitely no”. Differences were resolved by a separate researcher.

### Statistical analysis

The Stata 14.0 was used for data analysis. Dichotomous variables were presented with a risk ratio (RR) and 95% confidence interval (CI). For continuous variables, standardized mean differences (SMD) and 95% CI were calculated. The heterogeneity of included trials was assessed by *Q* and *I*^*2*^ statistics. As for Q statistics, *P* < 0.05 represented high heterogeneity. For *I*^*2*^ statistics, *I*^*2*^ < 25% indicated no significant heterogeneity, *I*^*2*^ = 25–50% represented moderate heterogeneity, and *I*^*2*^ > 50% showed high heterogeneity. When significant heterogeneity existed among included studies (*I*^*2*^ > 50%, *P* < 0.05), the random effects models were conducted. Otherwise, the fixed effects models were adopted. The subgroup analysis was performed according to various treatment durations and treatment strategies between groups. We hypothesized that the difference between ASC plus routine treatment and routine treatment alone would be larger than that between ASC and routine treatment. We also hypothesized that a longer treatment duration would show a larger difference in baseline levels compared with a shorter treatment duration. Tests of interactions were used to detect whether there were significant differences between subgroups and the five criteria were utilized to assess the credibility of significant subgroup effects [21]. Assessment of publication bias is generally only performed when 10 or more studies are included in a meta-analysis, thus it was not assessed as only 9 RCTs were included, and most analyses contained too few studies for the outcomes to undertake this.

### Assessing the certainty of the evidence–GRADE

The Grading of Recommendations Assessment, Development and Evaluation (GRADE) approach [22] was adopted to assess the certainty of the evidence for the review outcomes with five categories including risk of bias, imprecision, inconsistency, indirectness, and publication bias. The certainty of the evidence for each outcome measure was rated as “high”, “moderate”, “low” or “very low”.

## Results

### Study selection

**Fig. 1:**
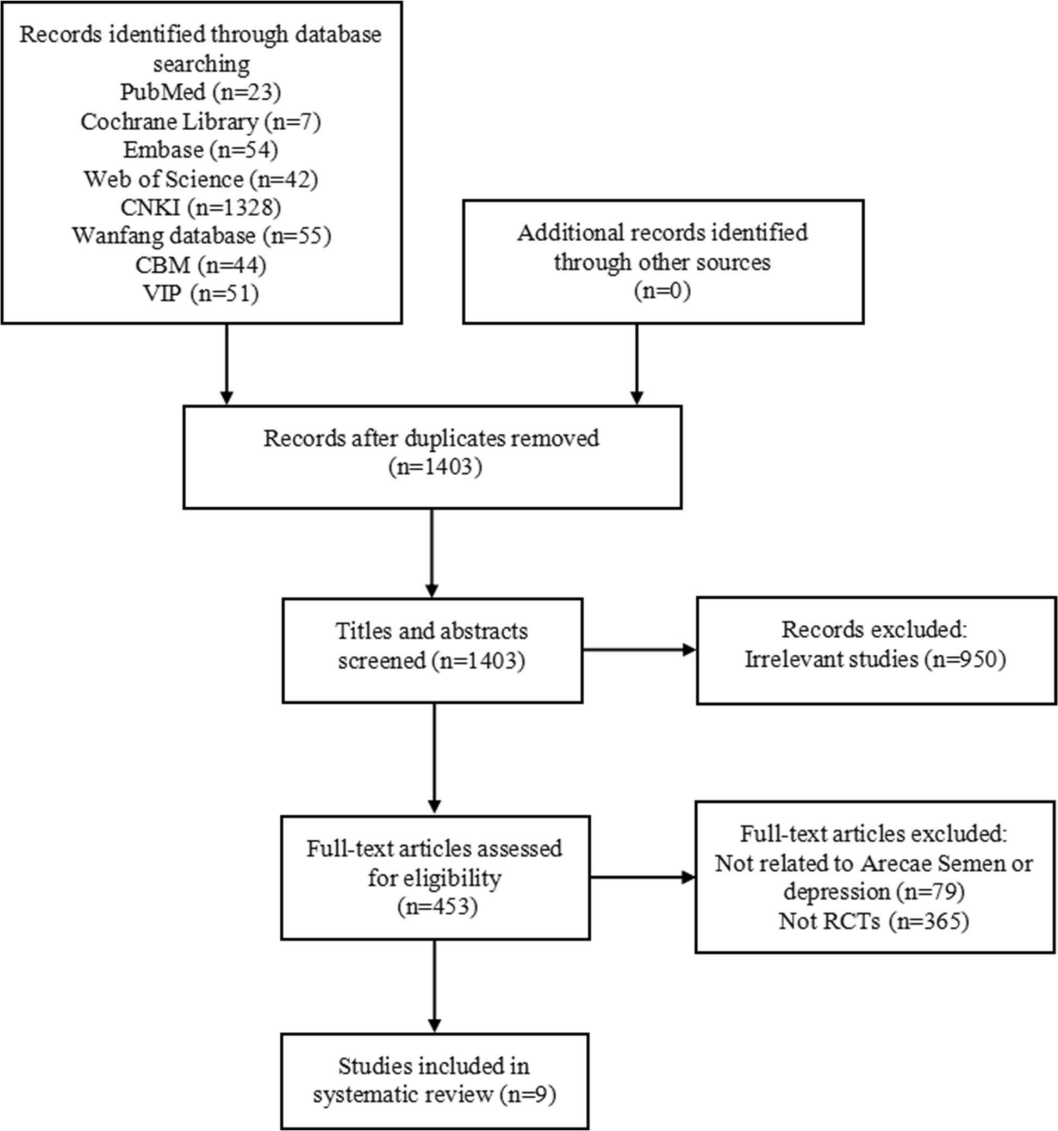
The flowchart of the study selection process

We identified 1604 potentially relevant articles after the initial search, of which 201 articles were removed owing to duplicate publication. After screening the title and abstracts, 950 articles were excluded because they were irrelevant studies. Of the remaining 453 articles, 444 articles were excluded by reading full texts, of which 79 were not related to Arecae Semen or depression, and 365 were non-RCTs. Finally, nine RCTs [16, 17, 23–29] were included in this review. The flowchart of the study selection process is shown in Fig. 1.

### Study characteristics

Nine RCTs [16, 17, 23–29] involving 787 patients were included in this review, of which 393 patients received ASC and formed the experimental group, and 394 patients did not receive ASC and formed the control group. Various comparisons included TCM versus routine treatment, TCM plus routine treatment versus routine treatment, and TCM plus acupuncture versus routine treatment. The included RCTs were conducted in China between 2008 and 2021. The treatment duration of included studies ranged from 2 to 8 weeks. The total effective rate was assessed in six RCTs [17, 23, 24, 26–28]. Only two studies [17, 25] did not evaluate adverse events. All studies reported HAMD scores [16, 17, 23–29]. Depression-related symptoms were reported in three studies [24, 26, 29]. The basic characteristics of all included studies are summarized in Table 3.

**Table 3:** Characteristics of included studies

Included trials	Year	Region	Study design	Sample size	Age (years)	Gender		Intervention	Control	Treatment duration	Outcome measures	Adverse events
						Male	Female					
Xue [16]	2014	China	RCT	185	I: 45.5 ± 7.5 C: 45.9 ± 7.3	I: 91 C: 94	/	Jieyu Xiere Decoction (one dose/day) + SSRIs	SSRIs + trazodone (50 mg/day)	4 weeks	①	TESS scores in the TCM group were lower than the control group, while there was no significant difference
Gu [17]	2021	China	RCT	104	I:43.59 ± 10.6 C:44.16 ± 10.48	I: 22 C: 24	I: 30 C: 28	Binglang Shisanwei Pill (9～13 pills, twice daily) +mental nursing	Agomelatine (25 mg/day) + usual care	2 weeks	①	Not mentioned
Nie [23]	2016	China	RCT	60	48.7 ± 2.9	21	39	Binglang Shisanwei Pill (10 pills, twice daily)	Fluoxetine (20 mg/day)	2 weeks	①	There were no adverse events such as liver function abnormalities, electrocardiography abnormalities, and constipation.
Liu [24]	2014	China	RCT	116	I: 37.2 ± 11.9 C:37.6 ± 12.5	I: 30 C: 28	I: 31 C: 27	Muxiang Shunqi decoction (one dose/day) + Escitalopram (10 mg/day for a week, then 20 mg/day)	Escitalopram (10 mg/day for a week, then 20 mg/day)	6 weeks	①②	The incidence of adverse events was lower in the TCM group compared with the control group (*P* < 0.05).
Zhang et al. [25]	2016	China	RCT	100	I:43.20 ± 10.09 C:42.50 ± 9.17	I: 22 C: 23	I: 28 C: 27	Yudian decoction (one dose/day)	Fluoxetine (20 mg/day, then increased to 20 mg twice daily)	4 weeks	①	Not mentioned
Huang et al. [26]	2021	China	RCT	78	I: 35.28 ± 6.61 C: 35.08 ± 5.83	I: 11 C: 10	I: 20 C: 21	Yueju Baohe pill (6 g, twice daily)	Amitriptyline (an initial dose of 25 mg twice daily, then showed an increase of 25–50 mg daily, eventually 100–175 mg/day)	8 weeks	①②③	The TESS scores were significantly lower in the TCM group compared with the control group (*P* < 0.001)
Pan et al. [27]	2008	China	RCT	24	Ranged from 18 to 60	I: 5 C: 4	I: 8 C:7	Yueju Baohe pill (6 g, twice daily)	Fluoxetine (20 mg/day)	4 weeks	①	No adverse events
Mi et al. [28]	2011	China	RCT	60	I: 62.8 C: 62.3	I: 18 C: 13	I: 12 C:17	Yueju Baohe pill (6 g, twice daily)	Fluoxetine (20 mg/day)	8 weeks	①	No adverse events
Zhou et al. [29]	2012	China	RCT	60	I:Ranged from 26 to 64 C: Ranged from 25 to 63	I: 12 C: 14	I: 18 C:16	Kuaiwei Shugan Pill (one dose, twice daily) +Fluoxetine (20 mg/day)	Fluoxetine (20 mg/day)	4 weeks	①②	There were fewer adverse events in the TCM group compared with the control group

*RCT* randomized controlled trials, *I* intervention group, *C* control group, *TESS* Treatment Emergent Symptom Scale, *TCM* Traditional Chinese Medicine, *SSRIs* Selective serotonin reuptake inhibitors, the most popular antidepressant medications. ①Hamilton depression rating scale (HAMD); ②depression-related symptoms; ③serum dopamine levels

### Risk of bias and certainty of evidence

**Fig. 2:**
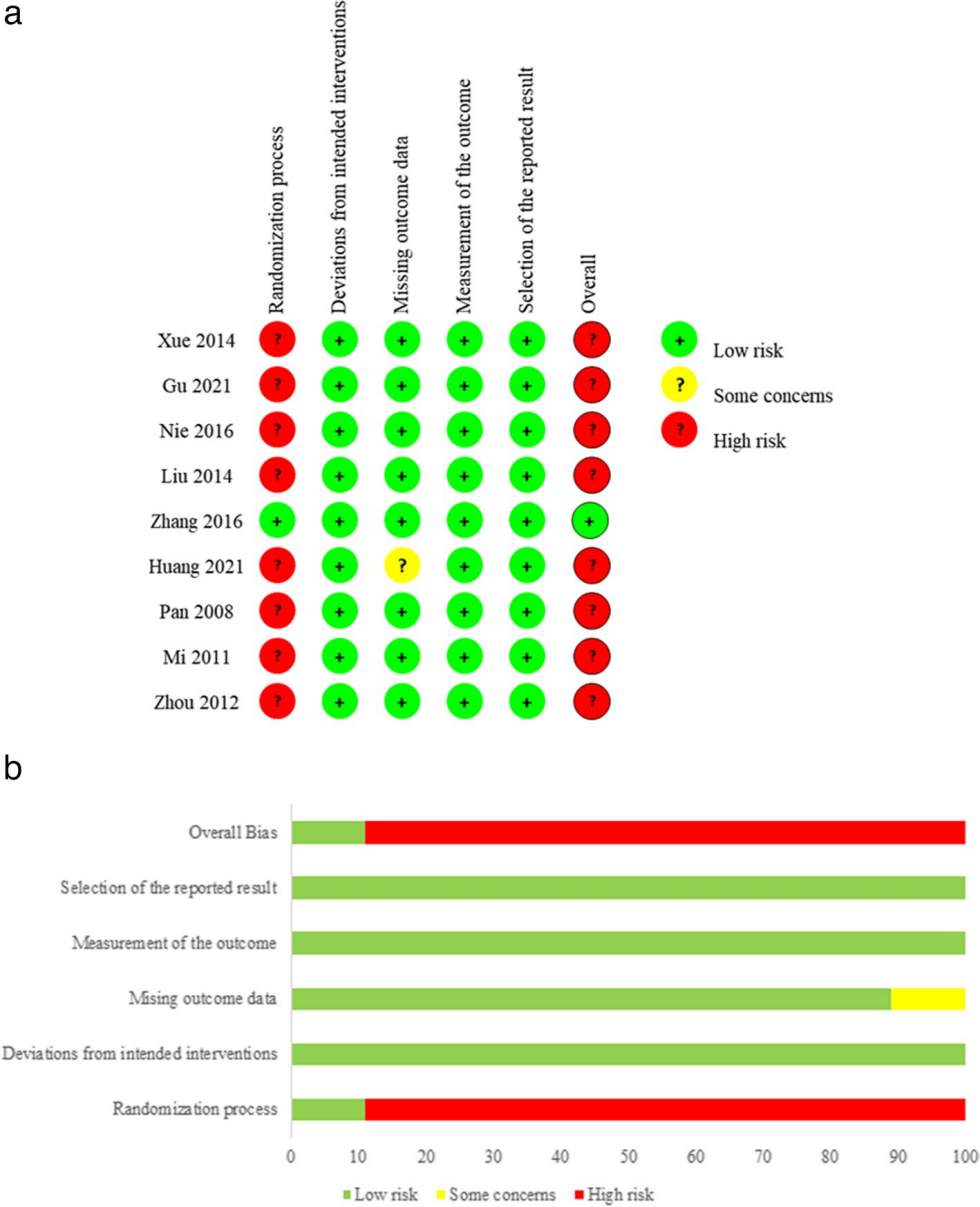
**a **Risk of bias of the studies included in the systematic review. **b **Overall risk of bias, with each category presented as percentages

The methodology quality of the included studies was assessed using the ROB 2, and the results are shown in Fig. 2a, b. All included studies mentioned randomization, but only four of them [17, 24, 26, 29] reported their randomization method, i.e., random number table. Three trials [16, 25, 27] allocated patients based on the admission time, and two trials [23, 28] did not provide detailed randomization methods. Only one trial [25] mentioned allocation concealment, thus the response option was “definitely yes”. The remaining eight trials [16, 17, 23, 24, 26–29] didn’t describe allocation concealment methods, which were judged as high risk of bias with response options “definitely no”. As for blinding of patients and healthcare providers, all studies were assessed to be at high risk of bias. Regarding data collectors, outcome assessors, and data analysts, the response options were “probably no”. All included trials showed a low risk of bias in incomplete outcome data and selective reporting. The GRADE evidence is presented in Table 4. The confidence of evidence was lowered owing to the risk of bias, inconsistency of results, and imprecision. The evidence of outcomes was of very low certainty.

**Table 4:** Quality of evidence in included studies with GRADE approach

Quality assessment						Summary of findings			
Number of studies (design)	Risk of bias	Inconsistency	Indirectness	Imprecision	Publication bias	Number of patients		Effect (95% CI)	GRADE quality
						TCM	Control		
HAMD scores									
9 (RCT)	Serious risk of bias ^a^	Serious inconsistency ^b^	No serious indirectness	Serious impression ^c^	Serious	390	390	SMD − 3.43, [− 5.24, − 1.61]	⨁○○○ Very low
TESS scores									
3 (RCT)	Serious risk of bias	Serious inconsistency	No serious indirectness	Serious impression	Suspected ^d^	157	159	WMD − 1.43, [− 2.58, − 0.29]	⨁○○○ Very low
Incidence of adverse events									
4 (RCT)	Serious risk of bias	No serious inconsistency	No serious indirectness	Serious impression	Suspected	129	118	RR 0.18, [0.04, 0.77]	⨁○○○ Very low

*TCM* Traditional Chinese Medicine, *CI* confidence interval, *HAMD* Hamilton depression rating scale, *TESS* Treatment Emergent Symptom Scale, *RCT* randomized controlled trials, *SMD* standardized mean differences, *WMD* weighted mean differences, *RR* risk ratio

^a^Allocation concealment cannot be achieved

^b^High *I* square

^c^Small sample size and wide boundary of the CI

^d^Only few studies

### Effectiveness assessment

#### HAMD scores

**Fig. 3:**
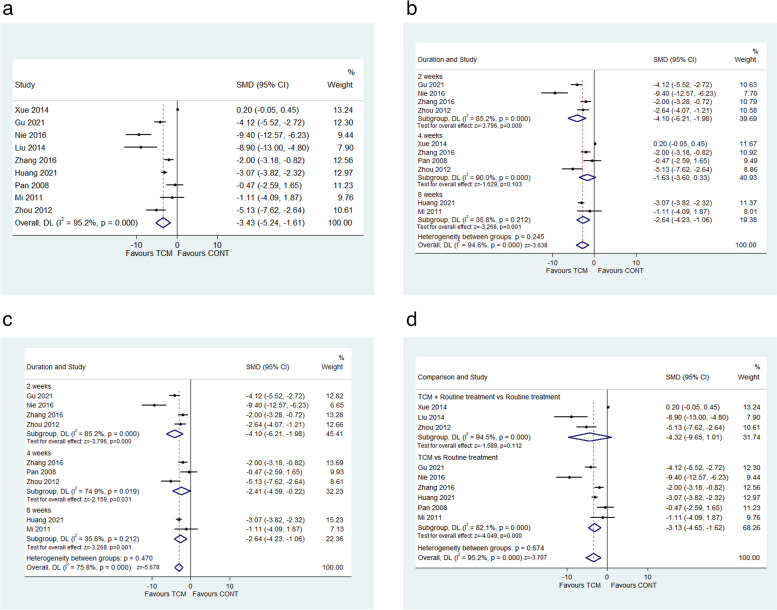
**a **The forest plot of HAMD scores at the longest follow-up. **b** The forest plot of HAMD scores at different follow-ups. **c **The forest plot of HAMD scores at different follow-ups after excluding one study probably leading to heterogeneity. **d **The forest plot of HAMD scores at different treatment strategies

Nine included studies [16, 17, 23–29] evaluated the effect of ASC on HAMD scores. There was high heterogeneity among these trials (*I*^2^ = 89.4%, *P* < 0.001), therefore a random effect model was utilized. The results indicated that ASC significantly decreased HAMD scores compared with the antidepressants (SMD − 3.43, 95% CI − 5.24 to − 1.61; *I*^2^ = 95.2%, *P* < 0.001; Fig. 3a). The minimal clinically important difference (MCID) was a minimum threshold introduced to reasonably explain the clinical significance of the changes in scale scores. The changes in HAMD scores were larger than the previously published MCID of HAMD scores of 1.3 [30], and the difference was clinically significant. Therefore, it could be interpreted that ASC may improve depressive disorders in patients with depression but the evidence is very uncertain.

The subgroup analysis was performed according to different durations of treatment and treatment strategies. Patients with depression receiving TCM intervention had a better effect on HAMD scores than those undertaking antidepressants in the 2-week follow-up subgroups (SMD − 4.10, 95% CI − 6.21 to − 1.98; *I*^2^ = 85.2%, *P* < 0.001). The TCM intervention showed similar reductions in HAMD scores at 8 weeks (SMD − 2.64, 95% CI − 4.23 to − 1.06; *I*^2^ = 35.8%, *P* = 0.001). The ASC for 4 weeks displayed clinical improvements in HAMD scores compared with the control group, but there was no significant difference (SMD − 1.63, 95% CI − 3.60 to 0.33; *I*^2^ = 90.0%, *P* = 0.103; Fig. 3b). We excluded one study [16] considering the subjects only limited to male patients, and the subgroup analysis was re-performed to remove the potential bias of baseline characteristics. The results indicated that patients with depression receiving TCM intervention could significantly lower HAMD scores than those undertaking antidepressants at 2 weeks (SMD − 4.10, 95% CI − 6.21 to − 1.98; *I*^2^ = 85.2%, *P* < 0.001), 4 weeks (SMD − 2.41, 95% CI − 4.59 to − 0.22; *I*^2^ = 74.9%, *P* = 0.031), and 8 weeks (SMD − 2.64, 95% CI − 4.23 to − 1.06; *I*^2^ = 35.8%, *P* = 0.001; Fig. 3c). When it came to the treatment strategies, 3 trials [16, 24, 29] compared the TCM therapy plus routine treatment with routine treatment, while no difference was found between the two groups in HAMD scores (SMD − 4.32, 95% CI − 9.65 to 1.01; *I*^2^ = 94.5%, *P* = 0.112). Six studies [17, 23, 25–28] evaluated the effects of TCM alone versus routine treatment, indicating that TCM therapy had a better effect on HAMD scores compared with routine treatment (SMD − 3.13, 95% CI − 4.65 to − 1.62; *I*^2^ = 82.1%, *P* < 0.001; Fig. 3d). There were no significant interactions according to duration of treatment (*P* = 0.245) and treatment strategies (*P* = 0.674) that chance could explain the subgroup difference. The subgroup difference has very low credibility based on the five criteria (Table 5).

**Table 5:** Criteria for assessing the credibility of significant subgroup effects

Criteria	Answer	Explanations
Can chance explain the subgroup difference?	Yes	*P* > 0.10 for the test of interaction
Is the subgroup difference consistent across studies?	No	High heterogeneity was represented in each subgroup
Was the subgroup difference one of a small number of a priori hypotheses in which the direction was accurately prespecified?	Yes	Priori hypotheses were pre-specified
Is there a strong preexisting biological rationale supporting the apparent subgroup effect?	No	We assumed that TCM plus routine treatment is superior to TCM therapy compared with routine treatment alone. The longer the treatment duration is taken, the effectiveness is better. However, there is no biological rationale supporting the apparent subgroup effect
Is the subgroup difference suggested by comparisons within rather than between studies?	No	These were comparisons between studies

#### Depression-related symptoms

Three RCTs [24, 26, 29] reported common depression-related symptoms, including pessimism, irritability, and sleep disorder. Only one study [24] used quality of life scores to evaluate the current symptoms tolerance, showing that ASC assessed higher quality of life scores than Escitalopram therapy. Furthermore, we were unable to perform a meta-analysis because two studies [26, 29] assessed symptom scores not based on the same criteria, one of which [26] extracted typical symptoms from the HAMD scoring system and calculated accumulated symptom points, while the remaining study [29] reported total TCM syndrome scores. Regarding decreasing symptom scores of depression, pessimism, dysphoria, and sleep disorders, TCM intervention was superior to routine treatment (*P* < 0.001) [24]. Compared with the control group, the TCM intervention significantly lowered the total TCM syndrome scores at 2 months and 4 months (*P* < 0.05) [29]. In this case, ASC showed beneficial effects on the improvement of depression-related symptoms.

#### Serum dopamine levels

Only one study [26] assessed serum dopamine levels, indicating that depressive patients with ASC significantly increased serum dopamine levels in comparison with the conventional treatment group (*P* < 0.01).

#### Adverse events

**Fig. 4:**
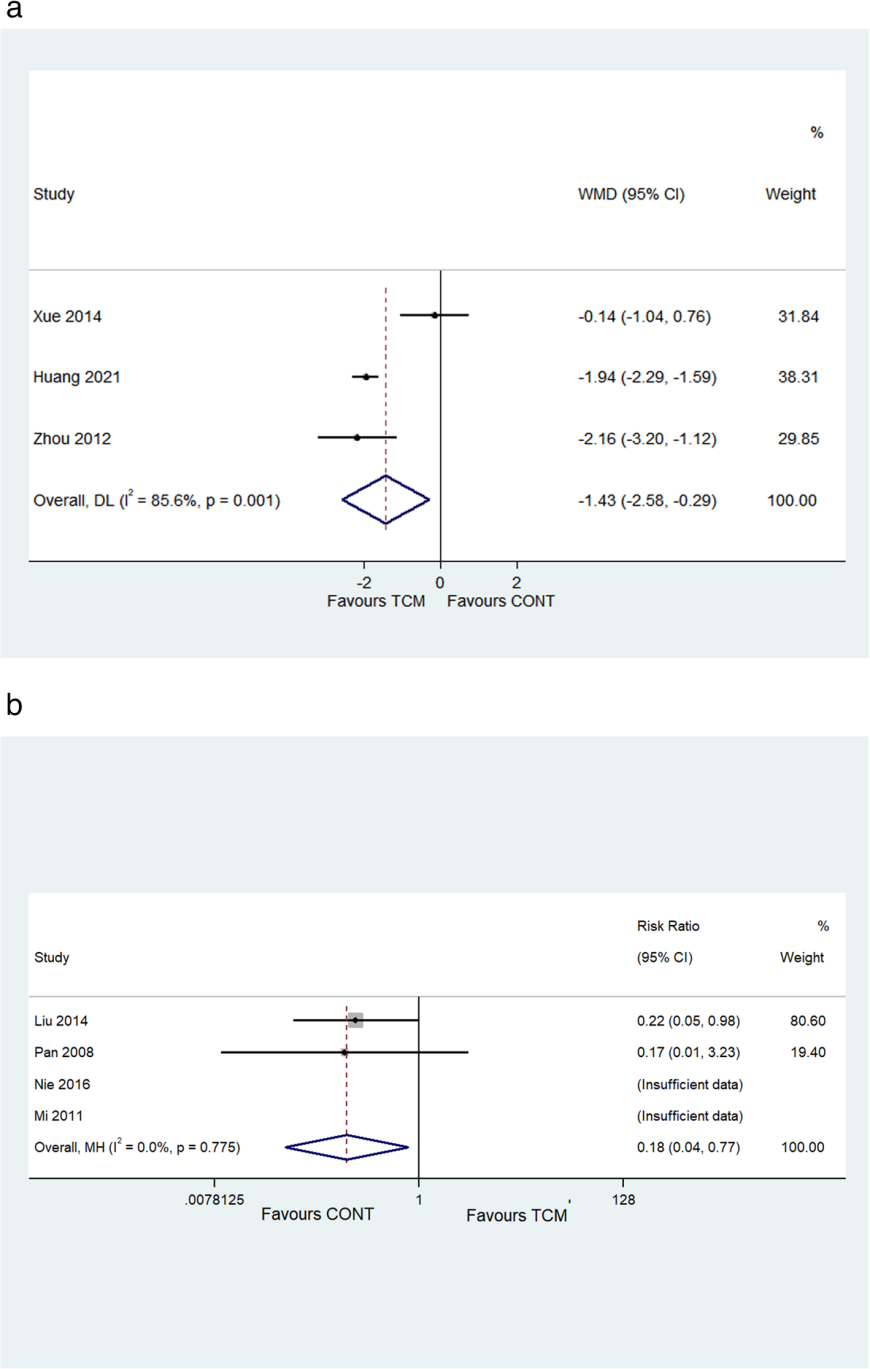
**a **The forest plot of TESS scores at the longest follow-up. **b **The forest plot of adverse events

A total of seven included studies [16, 23, 24, 26–29] reported adverse events, of which three RCTs [16, 26, 29] assessed the Treatment Emergent Symptom Scale (TESS). The random effect model was applied due to the high heterogeneity (*I*^2^ = 96.3%, *P* < 0.001). The results showed that the TCM intervention had lower TESS scores than the control group but the evidence is very uncertain (WMD − 1.43, 95% CI − 2.58 to − 0.29; *I*^2^ = 85.6%, *P* = 0.001; Fig. 4a). Adverse events were calculated in four trials [23, 24, 27, 28]. The combined effects indicated that TCM intervention was associated with a reduction in the incidence of adverse events, while there was no significant difference (RR 0.18, 95% CI 0.04 to 0.77; *I*^2^ = 0, *P* = 0.775; Fig. 4b) (adverse events were not observed in both TCM group and the control group in two studies [23, 28], thus these two studies were not included in the meta-analysis with the note of “insufficient data”). One trial [24] described that the adverse reactions of nausea and dry mouth observed in the TCM intervention group were significantly fewer in comparison with those of the control group. Three trials [23, 27, 28] mentioned that there were no adverse events in the TCM intervention group. In summary, the TCM intervention may decrease the incidence of adverse events but the evidence is very uncertain, indicating the potential safety of TCM intervention.

### Meta-regression analysis

**Fig. 5:**
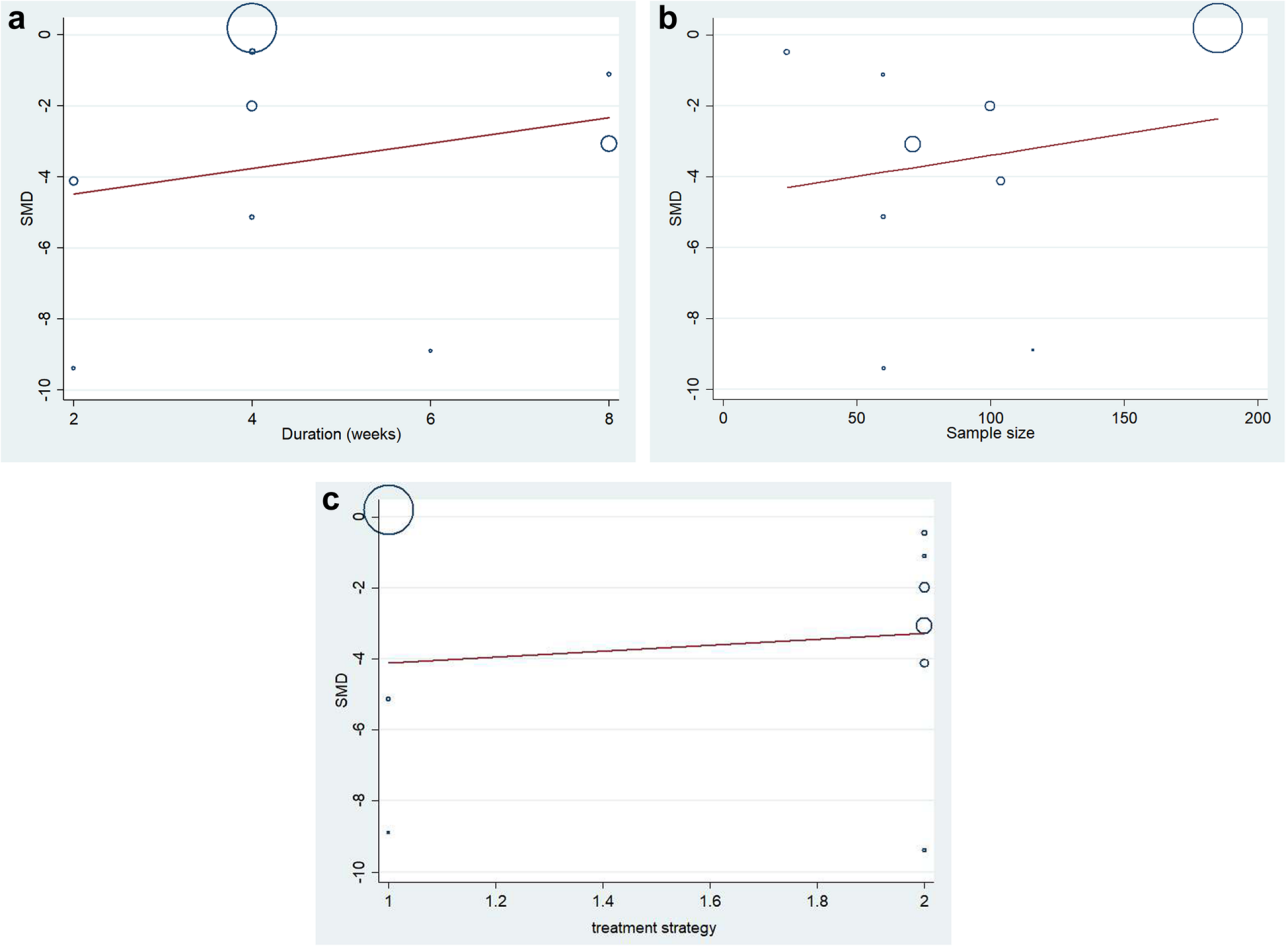
**a **Average HAMD scores during the overall duration.** b **Average HAMD scores for sample size of each included study.** c** Average HAMD scores for treatment strategy

The heterogeneity of HAMD scores was not associated with duration (slope 1.43, 95% CI 0.389 to 5.263, *P* = 0.537; Fig. 5a), sample size (slope 1.012, 95% CI 0.95 to 1.08, *P* = 0.658; Fig. 5b) and treatment strategy (slope 2.30, 95% CI 0.006 to 940.352, *P* = 0.753; Fig. 5c).

## Discussion

This review showed that compared with conventional therapy, ASC could reduce HAMD scores, alleviate depression-related symptoms, increase serum dopamine levels, and lead to fewer adverse events, but the evidence is very uncertain. This suggested that ASC might be an effective complementary and alternative therapy for patients with depression.

### Summary of evidence of TCM for depression

As far as we know, this study is the first systematic review to evaluate the effectiveness and safety of ASC for the management of depression. The evidence is very uncertain about the effect of ASC on HAMD scores, TESS scores, and incidence of adverse events. The findings of this review suggested that compared to the control group, ASC exerted better effects on lowering HAMD scores (SMD − 3.43, 95% CI − 5.24 to − 1.61; *I*^2^ = 95.2%) as well as TESS scores (WMD − 1.43, 95% CI − 2.58 to − 0.29; *I*^2^ = 85.6%), either as an adjunct or replacement for conventional therapy. The results were similar to the previous study, which validated the anti-depressive effects of TCM intervention [31]. The subgroup analysis of HAMD scores was performed due to the significant heterogeneity, indicating that the TCM group had an advantage over the control group at different treatment durations. Moreover, TCM therapy combined with routine treatment was more beneficial in HAMD scores than routine treatment alone, while no differences were found between the two groups (*P* = 0.112). The TCM therapy showed a significant effect on decreasing HAMD scores compared with routine treatment (*P* < 0.001). The sample size, treatment duration, and treatment strategy did not adequately account for the heterogeneity. The results of this review supported evidence from previous research [32], which demonstrated that TCM therapy in China could significantly improve depressive symptoms and decrease HAMD scores in combination with antidepressants.

### Mechanisms of Arecae Semen for depression

Increasing evidence has investigated that Arecae Semen possesses antidepressant ability. The results from a phytochemical analysis have revealed that saponins of Arecae Semen may be the active components against depression, which may be associated with the elevation of serotonin and noradrenaline [33]. According to experimental research, the Areca catechu nut treatment may ameliorate depressive symptoms by promoting the demyelination process in the prefrontal cortex [34]. The latest research has found that the potential antidepressant mechanisms of Arecae Semen may be related to increasing monoamine neurotransmitters in the brain and regulating oxidative stress [35]. An experimental study has highlighted the critical role of neurotrophic signaling that Areca Thirteen Pill might exert antidepressant actions through regulating the cAMP/PKA/CREB/BDNF signaling pathway, increasing proliferative activity, and suppressing apoptosis [36].

Grounded on the meridian theory, Arecae Semen follows the liver meridian, as recorded in Bencao Jingjie (Interpretation of Classic of Materia Medica) written by Ye Gui in the Qing Dynasty. A lot of medical records state that Arecae Semen is nontoxic and can activate qi flowing of the liver meridian. TCM theory holds that liver qi depression may be the primary pathogenesis of mental disorders. Besides, a deficiency of spleen qi may affect the function of the liver for controlling conveyance and dispersion, resulting in liver depression. Therefore, it is crucial to promote the circulation of qi and relieve stagnation in patients with depression. ASC is formulated based on the principle of dispersing stagnated liver qi and removing the obstruction, which is beneficial for patients who suffer from depression. Danxi Xinfa (Danxi’s Mastery of Medicine) has recorded that Yueju Pill, an effective ASC, can achieve curative effects in patients with depression. As shown in the included trials, Arecae Semen is usually compatible with Rhizoma Cyperi, Radix Aucklandiae, Fructus Aurantii Immaturus, and so on, therefore enhancing the effects of activating qi. The TCM compatibility exerts pharmacological effects through multiple targets and pathways. There is some clinical evidence that ASCs are commonly applied to patients with depression.

### Medicinal and edible Arecae Semen

Increasingly studies have paid attention to the safety of Arecae Semen, claiming that long-term consumption is addictive, may have carcinogenicity, and cause damage to multiple organ systems [37]. However, there are great differences between medicinal and edible Arecae Semen in materials, processing technology, and edible methods [38].

Medicinal Arecae Semen refers to the mature seed of the areca fruit, and edible Arecae Semen uses the young fruit, including the shell and skin. Edible Arecae Semen is soaked with limewater, and then strongly alkaline and highly irritating essences or spices are added. These auxiliary materials have excitatory effects and also contain carcinogenic substances which may lead to oral mucosal injury. Medicinal Arecae Semen must experience a range of procedures including processing, extraction, and impurities removal, producing obvious detoxification effects. Essences or spices are not added to medicinal Arecae Semen. Edible Arecae Semen is often chewed for several hours, making crude fiber and hard fruit core repeatedly rub and stimulate the oral mucosa, which may cause oral premalignant lesions such as submucosal fibrosis, leucoplakia, and moss, and eventually result in the occurrence of oral cancer [39]. Medicinal Arecae Semen is taken orally in decoction, without long-term irritant to oral mucosa. In addition, there are differences in the dosage of medicinal and edible Arecae Semen. Edible Arecae Semen has no limits on dosage, thus it’s often consumed in large quantities. The Chinese Pharmacopeia 2020 edition [40] has prescribed the dosage of medicinal Arecae Semen ranging from 3 to 10 g, and when it produces effects on expelling parasite, the dosage can be increased to the range of 30 to 60 g. The preceding dosages of medicinal Arecae Semen are safe in clinical practice. Chewing edible Arecae Semen is a common life custom from the Asia–Pacific region, and tends for chronic and accumulated injury. In contrast, with the short course of treatment and low-dose application, the medicinal Arecae Semen will not induce toxicity, chronic injury, or precancerous lesions. Oral cancer induced by edible Arecae Semen consumption involves physical and chemical factors, dosage, course of treatment, usage, and so on. The combination of all these negative factors may contribute to an increase in the incidence of oral cancer. But unlike edible Arecae Semen, effective measures in all aspects are carried out to ensure the safe application of medicinal Arecae Semen.

In brief, there are obvious distinctions between medicinal and edible Arecae Semen. The unlimited use of edible Arecae Semen is prone to suffer from oral cancer, while a number of steps have been taken on medicinal Arecae Semen. According to the compatibility and dosage of medicinal Arecae Semen recorded in the Chinese pharmacopeia, few adverse events have been reported so far, thus there is no evidence for carcinogenicity of medicinal Arecae Semen.

### Safety of Arecae Semen’s clinical use

Prepared by processing and frying, medicinal Arecae Semen is compatible with other herbs to exert its pharmacological effects. Besides, active ingredients of medicinal Arecae Semen are extracted and made into a variety of Chinese patent medicine products. The Chinese Pharmacopoeia 2020 edition [40] neither defines Arecae Semen as a toxic herb nor states any warnings of its careful clinical application. The reported toxicity has been concentrated on products of edible Arecae Semen as well as the diverse chemical ingredients primarily areca alkaloids [41–43]. Medicinal Arecae Semen is widely used with few adverse events observed, which may be associated with toxin reduction owing to processing and formula compatibility of TCM. There are a few disputes about the safety of medicinal Arecae Semen in practical clinical application, which cannot be confused with the risks of long-term chewing edible Arecae Semen.

Though there is no evidence of medicinal Arecae Semen causing oral cancer, it still has some adverse events. Ancient literature has documented some adverse events of medicinal Arecae Semen. For example, Shiliao Bencao (Materia Medica for Dietotherapy) and Bencao Huiyan (Collected Works of Materia Medica) recorded that overuse of medicinal Arecae Semen might contribute to fever and draining of strength respectively. Current researchers have reported that an overdose of ASC can develop symptoms such as nausea, sweats, abdominal pain, heart palpitations, and so on [44, 45]. Therefore, medicinal Arecae Semen has good safety without causing oral cancer, though sometimes gastrointestinal or neurological symptoms can be observed.

### Strengths and limitations

The novelty of this review lies in highlighting the effects of ASC on depression. As a widespread tradition in Southeast Asia, areca nut chewing is an important risk factor for oral cancer and oral submucous fibrosis. Most researches focus on the carcinogenicity of Arecae Semen, and only a few studies have explored the medicinal values of Arecae Semen. This review summarized clinical evidence of TCM for depression and demonstrated the values of Arecae Semen from the perspective of pharmacology and TCM theory. In addition, the GRADE tool was developed to assess the quality of evidence.

However, several limitations are supposed to be taken into account when interpreting these results. First, no included trials have their protocols registered. Clinical trial registration can ensure research transparency and reduce the risk of bias. Second, all included studies reported randomization, while three of them provided detailed randomization according to the admission time, and two studies did not describe the concrete randomization methods. Most of the included trials did not mention allocation concealment. These might lead to selection bias. Third, the confounding factors, such as sample size, treatment duration, and compositions of Chinese herbal prescriptions varied between studies. In this case, the results should be interpreted with caution due to the limited methodology quality. Fourth, all included trials were performed in China, which might result in publication bias.

This review has summarized the latest evidence for ASC on depression, which provides a direction for further clinical application on usage and compatibilities of Arecae Semen among patients with depression. More experimental researches are required to fully investigate the actions of Arecae Semen. Further large-scale and rigorous-designed RCTs are warranted to validate the results of this review. This review also discussed the differences between medicinal and edible Arecae Semen, thus it is necessary to strengthen market supervision of edible Arecae Semen and its products. Moreover, measures should be taken to develop extended products on medicinal Arecae Semen to maintain the industrial chain. Laws and regulations on the quality, dosage, and safety of medicinal Arecae Semen need to be published to ensure its reasonable and safe application and dispel global misunderstanding of safety.

## Conclusions

In general, ASC may lower HAMD scores, alleviate depression-related symptoms, increase serum dopamine levels, and reduce the incidence of adverse events, which provides an effective and safe therapy for patients with depression. The results should be interpreted with caution owing that the evidence is very uncertain. Future researches are required to verify our results and further investigate the medicinal value of Arecae Semen.

## Supplementary Information

Additional file 1: PRISMA 2020 Checklist.

## Data Availability

The data used to support the findings of this study are available from the corresponding author upon request.

## Abbreviations

TCM: Traditional Chinese medicine
ASC: Arecae Semen compounds
RCTs: Randomized controlled trials
HAMD: Hamilton Depression rating scale
GRADE: Grading of Recommendations Assessment, Development and Evaluation
RR: Risk ratio
CI: Confidence interval
MCID: Minimal clinically important difference
TESS: Treatment Emergent Symptom Scale
